# Inherited retinal disorders in Scotland: A 5 year assessment

**DOI:** 10.1038/s41433-025-04216-z

**Published:** 2026-01-09

**Authors:** James E. Hazelwood, Mertcan Sevgi, Fiona Osborne, Blazej Staniszewski, Nick George, Andreea Ionean, David F. Gilmour, Roly Megaw

**Affiliations:** 1 Princess Alexandra Eye Pavilion, NHS Lothian, Chalmers St, Edinburgh, EH3 9HA UK; 2 The University of Edinburgh, Edinburgh, UK; 3 Gartnavel hospital, NHS Greater Glasgow & Clyde, Aberdeen, UK; 4 Aberdeen Royal Infirmary, NHS Grampian, Aberdeen, UK; 5 Ninewells hospital, NHS Tayside, Aberdeen, UK; 6 MRC Human Genetics Unit, MRC Institute of Genetics & Cancer, University of Edinburgh, Western General Hospital, Edinburgh, EH4 2XU UK

**Keywords:** Hereditary eye disease, Retinal diseases

## Abstract

**Objective:**

Inherited Retinal Disorders (IRDs) are a leading cause of blindness in working age adults. With the emergence of therapeutic approaches and pre-implantation genetic testing, obtaining a molecular diagnosis is increasingly important. As such we aimed to determine the caseload of IRD patients presenting to Scottish ophthalmology services over a 5-year period, and evaluated the diagnostic approaches used and results of genetic testing in order to determine any established inherited cause.

**Subjects:**

Data from all patients presenting to ophthalmology services across Scotland from January 2018 to January 2023 diagnosed with an IRD were collected. History and examination findings were recorded, as were the methods of genetic testing used, the identified genetic variants and the time taken for molecular reporting.

**Results:**

532 patients were included in the analysis. The most common clinical diagnosis was retinitis pigmentosa (RP) (42.4%), followed by Stargardt Disease (9.0%), with Usher syndrome the most common syndromic RP. The most common initial test was a 176 gene panel, followed by direct testing for ABCA4, with different regions pursuing different testing strategies. 67.4% of patients received a molecular diagnosis. The most common causal gene was ABCA4, followed by USH2A and RDS/PRPH2.

**Conclusion:**

This study provides the first assessment of IRDs in Scotland over a 5 year period. We demonstrate that the most common clinical diagnoses are RP and Stargardt disease, and that the majority received a molecular diagnosis. This work provides a unique insight into the Scottish ophthalmic genetics service and serves as a benchmark for iterative improvement.

## Introduction

Inherited retinal disorders (IRDs) are a group of monogenic conditions that, collectively, are the leading cause of blindness registration in working age adults in England and Wales [1, 2]. Over 280 causative genes have been identified, mutations in which disrupt key molecular pathways in the retina that, ultimately, lead to photoreceptor degeneration and visual loss [3]. Collectively, they are reported to have an incidence of 1 in 4000 people [4].

Determining the molecular diagnosis is beneficial to patients for prognostication and family planning reasons, including the potential for pre-implantation genetic testing [5]. More recently, advances in gene-specific therapies have increased this need. The first gene therapy for IRDs to be approved by the FDA and EMA (in 2017 and 2018, respectively), *Voretigene neparvovec-rzyl* (Luxturna®), requires the presence of biallelic (likely) pathogenic *RPE65* variants for treatment eligibility, emphasising the importance of an early molecular diagnosis. Gene augmentation therapies for other IRDs are currently in clinical trial [6]. Thus, optimising the molecular diagnosis rate for IRDs is paramount.

In the UK, conventional panel testing (the curation of a set of genes known to be associated with the condition) has been a common approach for identifying the genetic cause of rare diseases [7, 8]. NHS Scotland has previously utilised the 176 gene panel developed by the Manchester Centre for Genomic Medicine for their testing. The advent of next-generation sequencing, however, has improved molecular diagnosis rates for a broad range rare diseases over the past decade, with studies by the 100,000 genomes project demonstrating that 14% of diagnoses made by whole genome sequencing (WGS) were identified in genomic regions that would be missed by more conventional panel testing approaches [9]. In 2022, in light of these findings (and in keeping with Genome UK’s vision [9]), NHS England, supported by Genomics England, implemented the world’s first whole genome sequencing service for adults and children with rare disease, including those with an IRD. Following a strategic review, the Scottish government instead opted to develop a panel test for IRDs [10]. To prospectively audit the effects of this change in testing, identifying the burden of IRDs in Scotland and the diagnosis rates prior to this switch is important. Furthermore, no previous work has described inherited retinal diseases on a Scottish population level. Notable large scale previous work has investigated inherited retinal diseases in a UK setting, including the 100,000 genome project [11–13]. However, Scotland’s complex and varied genetic ancestry, (including Norse, Celtic and Germanic) gives rise to increased haplotype diversity that is poorly represented in larger UK or global work [14]. Additionally, key founder mutations including the *C1QTNF* mutation of Late Onset Retinal Degeneration (LORD) have their origins in Scotland [15]. As such a better understanding of Scottish-specific IRD landscape is useful to optimise future service design and workflows.

To this end, we sought to survey the landscape of IRD investigation and diagnosis in the 5 years prior to Scottish-based testing. We determined the numbers of patients presenting to Scottish ophthalmology services with an inherited retinal dystrophy, their clinical diagnoses, the uptake for molecular testing, the causative genes identified and the genetic testing methods used in making these diagnoses.

## Methods

In this retrospective, observational study, data was collected for all patients presenting to the ocular-genetics clinics across Scotland from January 2018 to January 2023 with a diagnosis of an inherited retinal disorder. Patients attended one of the 4 tertiary referral centres in Edinburgh, Glasgow, Dundee and Aberdeen, covering their respective geographical locations. New patients, patients undergoing further testing having previously tested negative, or those repeatedly declining molecular testing were included in the analysis. Return patients investigated prior to 2018 were excluded, as were those with albinism, Stickler’s disease, Von Hippel Lindau, tuberous sclerosis, congenital anterior chamber dysgenesis, or inherited optic neuropathies. If present, duplicate records were excluded at each centre using patient identifiers, and partially complete records included for analysis. Data collected included demographics, decade of symptom onset, presence of family history or consanguinity, testing performed and result, and time to result. Institutional approval was obtained from NHS Lothian, and research adhered to the Declaration of Helsinki.

### Genetic testing

Patients underwent full ophthalmic examination including full clinical history and family history, with OCT, fundus autofluorescence and electrodiagnostic testing, as required. If an IRD was suspected, patients were offered genetic testing and investigated either using the 176 gene retinal panel (through the Manchester Centre for Genomic Medicine) or, if presenting with specific phenotypic signs, more targeted sequencing. Some of the targeted tests were able to be performed locally, including *ABCA4, C1QTNF, BEST, RS1 and RDS/PRPH2*. In appropriate cases, a negative targeted test was followed up by the 176 gene panel testing.

### Statistical analysis

Statistical analysis was performed using R Studio (4.3.2). Descriptive statistics explored patient and clinical demographics.

Chi-squared testing was used to assess differences between observed and expected frequencies of categorical variables, and Kruskal-Wallis and Dunn’s test with Bonferroni Correction was used to assess for significant mean differences between regions for elapsed time from test to result.

## Results

### Demographics

532 patients were included in the analysis: 224 male, 234 female and 74 with unrecorded sex. 262 patients were from the West of Scotland, 177 from Southeast Scotland, 20 from Tayside and 73 from Grampian. The mean age of patients was 57.4 years old, with the modal decade of onset of symptoms being the 3rd.

**Fig. 1:**
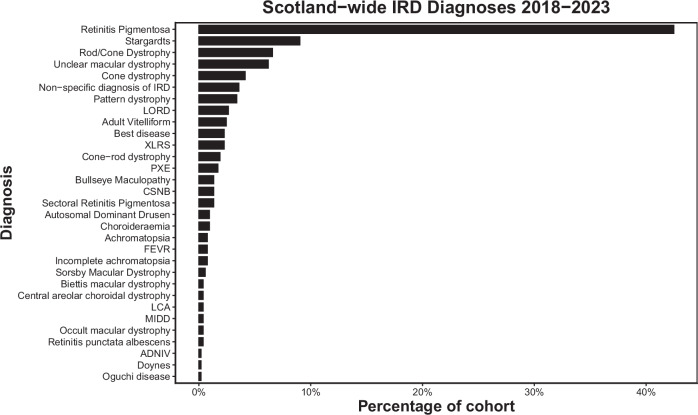
The frequency of diagnosis of Inherited Retinal disorders in Scotland 2018-2023. (IRD inherited retinal disorder, LORD late onset retinal degeneration, PXE Pseudoxanthoma elasticum, XLRS = X-Linked Retinoschisis, CSNB Congenital Stationary Night Blindness, FEVR Familial Exudative Vitreoretinopathy, MIDD Maternally Inherited Diabetes and Deafness, LCA Lebers Congenital Amaurosis, ADNIV Autosomal Dominant Neovascular Inflammatory Vitreoretinopathy.

**Fig. 2:**
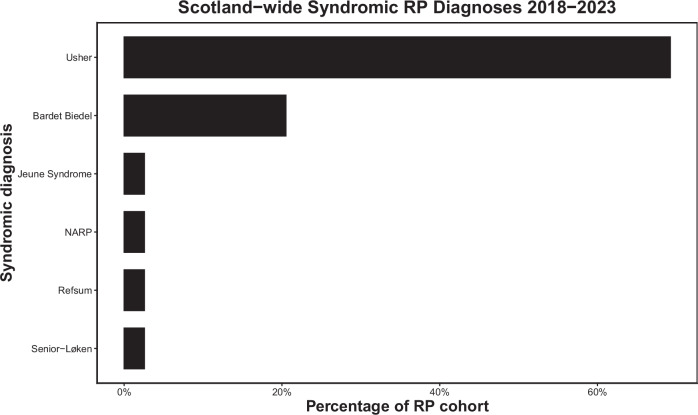
The frequency of diagnosis of types of syndromic retinitis pigmentosa, as a subset of total retinitis pigmentosa diagnoses in Scotland 2018-2023. (RP retinitis pigmentosa, NARP neuropathy, ataxia and retinitis pigmentosa).

The most common clinical diagnosis was Retinitis pigmentosa (42.4% *n* = 226), followed by Stargardt disease (9.0% *n* = 48) (Fig. 1, Supplementary Table S1). Usher syndrome was the most common syndromic RP (Fig. 2). For those with a known family history of retinal disease, the most common clinical diagnosis was RP, followed by late onset retinal degeneration (LORD). Of those with RP, 35 patients (15.5%) had a diagnosis of syndromic RP, with no significant difference in age at diagnosis between this group and those with simplex RP (*p* = 0.9617).

**Fig. 3:**
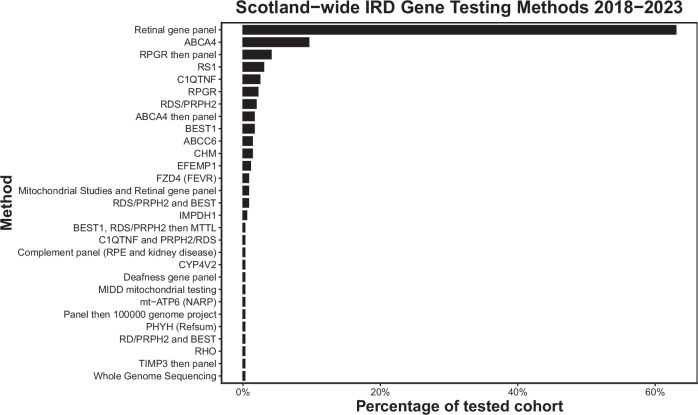
The frequency of the genetic testing methods used in the assessment of IRDs in Scotland 2018-2023. (IRD inherited retinal disorder).

27 patients (5.1%) with a clinical diagnosis of an IRD declined molecular testing. The most common initial testing method performed was Manchester’s IRD gene panel, followed by direct testing for *ABCA4*, *RPGR, RS1, C1QTNF*, and *RDS*/*PRPH2* (Fig. 3, Supplementary Table S2). Different regions appeared to have different testing strategies. The West of Scotland tested 72% of their patients with a gene panel initially, with other regions more likely to target a specific gene in the first instance (Table 1).

**Table 1:** The outcomes of genetic testing, by region. The time taken to receive a result from genetic testing, (from appointment at ocular genetics clinic), the percentage of initial investigations sent that were the 176 gene panel, and the percentage of tests sent that received a molecular diagnosis, in Scotland 2018-2023 by region.

Region	*N* =	Mean time to result (months)	SD	Percentage first test as panel	Percentage with molecular diagnosis
**Grampian**	57	7.77*	7.44	50	68.4
**Lothian**	111	7.14*	5.98	52.3	69.4
**Tayside**	7	8.17	7.68	47.3	85.7
**WOS**	190	4.08*	3.38	72.3^+^	65.2

(WOS West of Scotland, * significant mean difference to fastest region, WOS-Grampian *p* < 0.01; WOS-Lothian *p* < 0.01, ^+^*p* < 0.01).

**Fig. 4:**
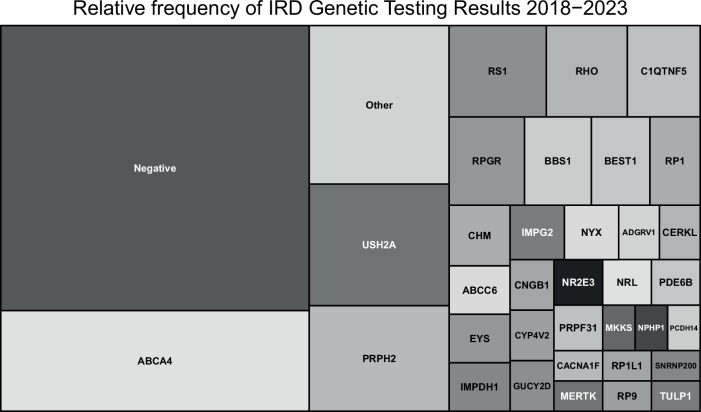
A treemap demonstrating the relative frequency of genetic testing results for inherited retinal disorders in Scotland 2018-2023, grouped by causal gene identified. Genes identified with a frequency of 1 are grouped as “Other”.

In 78 cases, the result was still outstanding, whilst 34 patients were awaiting their joint clinical genetics-ophthalmology appointment, at which point a decision would be made on testing. Of the 365 patients with a confirmed molecular diagnosis, 119 (32.6%) had no molecular diagnosis identified (‘negative’ in Fig. 4). 67.4% (246/365) of Scottish patients received a confirmed molecular diagnosis for their IRD. The most common causal gene identified was *ABCA4*, followed by *USH2A* and *RDS/PRPH2* (Fig. 4, Supplementary Table S3). 65.2% of patients undergoing a retinal gene panel received a molecular diagnosis (Supplementary Table S4). Targeted testing of *C1QTNF* confirmed pathogenic mutations in 88%, and *ABCA4* in 77% of cases. In several tests, 100% of cases received a molecular diagnosis– *BEST1, CYP4V2*, Deafness Panel, *PHYH, RHO, RS1, TIMP3, IMPDH1*, MIDD mitochondrial testing and *mt-ATP6*.

Rates of molecular diagnosis varied across the regions though was not significant (*p* = 0.21) (Table 1). Mean ‘time from testing to result’ varied between trusts, with Tukey analysis of means showing significant mean differences between WOS and Lothian (p < 0.001) and WOS and Grampian (*p* < 0.001) (Table 1). This could be explained by the WOS being significantly more likely to opt for a panel test in the first instance (72.3%, *p* < 0.01; Table 1), as negative results from targeted genes were mostly followed by a panel test.

## Discussion

This study presents a description of inherited retinal disorders in Scotland over a 5-year period. The most common initial testing strategy was a retinal gene panel, but this varied by region, with some more likely to attempt targeted testing in the first instance. Volume of genetic testing varied by region; a reflection of their respective populations. The most common clinical diagnoses were Retinitis pigmentosa and Stargardt disease, with the most common RP syndrome being Usher’s disease. The most common genes identified were *ABCA4*, *USH2A*, *RDS/PRPH2*, *RS1*, *RHO* and *C1QTNF*.

Previous work describes a range of molecular diagnosis rates in IRD. Khan et al. reported a molecular diagnosis rate of 40% in patients with IRD, and Ellingford described a 50% success rate [7, 16]. This is lower than the 67.4% reported in our study, but comparison of results is limited as their work related to the use of a 105 gene panel, rather than the 176 panel. Sheck et al.’s more recent work describes IRD molecular diagnosis rates in Moorfields between 2016 and 2018 using the 176 gene panel and reported a higher rate of 59.4% [8]. Further, they observed that a younger age at testing and certain diseases were more likely to result in a molecular diagnosis. In this study, diseases with a strong phenotype:genotype relationship initially underwent more targeted gene screening, and were thus not included for analysis. Our data includes analysis of targeted gene testing which had a slightly higher molecular diagnosis rate, increasing the overall molecular diagnosis rate in our cohort. Indeed, when analysed separately, our 176 gene panel results broadly agree, at 65.2%.

Previous work has demonstrated higher rates of molecular diagnosis in those presenting at a younger age. Taylor et al. report 78.8% of 85 cases under the age of 16 had a molecular diagnosis during a period of time when both 105 and 176 retinal gene panels were used [17]. Similarly, Sheck et al. reported 81.8% received a molecular diagnosis if <10 years old [8]. Our results show a similar trend, with 79.3% obtaining molecular diagnosis if a clinical diagnosis is made within first 2 decades. The reasons for this are likely multifactorial but could reflect that mutations with high functional consequences are more likely to be exonic and so easier to detect. Conversely, it is suggested that less pathogenic mutations (causing later onset disease) may be under less selective pressure and therefore of higher prevalence, making them harder to detect bioinformatically [8].

Regarding the most common clinical diagnoses, our work agrees closely with that reported from the My Retina Tracker initiative, a database of over 15,000 patients across the globe with IRDs. Similar to our work, they report that the most common diagnosis is Retinitis pigmentosa, followed by Stargardt disease and Usher syndrome [18]

Regarding the relative frequency of mutation in individual genes, our Scottish results are largely in keeping with the Global Retinal Inherited Disease dataset, who report that the most common mutation in the literature is *ABCA4*, followed by *USH2A* [19], with *RHO* and *PRPH2* also common. This is similarly reflected in work performed by Moorfields, but perhaps unsurprisingly, given the founder mutation originated in East Lothian [20], our Scottish cohort contains significantly more *C1QTNF5* mutations than national global studies [12].

The proportion of RP that is syndromic has previously been reported to range from 20-30% [21]. Our results broadly agree with this figure, but we report a slightly lower rate of syndromic RP at 15.5%, though rising to 19.4% (35/180) among RP cases with a molecular diagnosis. Our results are in keeping with other studies, whereby syndromic RP is most commonly diagnosed in patients with Usher syndrome, followed by Bardet-Beidl, then Refsum disease [22, 23].

Few previous studies have reported real world duration of genetic panel tests, particularly in the context of IRDs. Some studies have reported time to a molecular diagnosis of 12 to 21 weeks [24, 25]. The American Academy of Ophthalmology refers to the context of ‘clinically relevant’ turnaround time, noting that speed and cost increase in tandem and that 6 months would be a reasonable target [26]. Our mean turnaround time of 5.6 months is within this notional target, though the reasons for some regions taking longer will be reviewed in further work. When NHS England transitioned to offering whole genome sequencing (WGS) as part of routine care for monogenic disease, NHS Scotland instead opted to develop a targeted exome for testing IRDs [27]. Future work will aim to analyse the impact on speed and rate of diagnosis.

In our Scottish cohort, over 30% did not receive a molecular diagnosis. Whilst some of this missing heritability may be due to as yet undiscovered causal genes, it is known that adoption of WGS into IRD screening, with the resulting capture of 5’ prime and intronic sequences of known genes, can increase the proportion of cases obtaining a molecular diagnosis by 10-15% [9, 28, 29]. A national transition to this testing paradigm, matching NHS England, has significant potential for benefit. This has already been recognised; in their “Genomic Medicine Strategy 2024–2029” the Scottish Government articulate their ambition to update and develop their testing strategies to include WGS, and ensure that associated genomic data is incorporated into national datasets [30]. Aligned WGS approaches would also help to standardise IRD testing pathways, leading to reduced regional variation and improved time-to-diagnosis, with this work providing a useful baseline with which to measure improvement.

This study provides a comprehensive assessment of the ophthalmic genetics service across Scotland over a 5-year period, involving a large number of patients across all of the main ophthalmic centres. However, we are limited in the completeness of our assessment by the number of patients still to be seen in genetics clinic or awaiting their genetics results. Furthermore, as inclusion in the study was contingent on referral and clinic attendance in a tertiary centre, there may be a degree of under-representation of certain communities or districts if they are underserved or under-referred by community ophthalmic teams, As the period studied includes the COVID-19 era, there may be some artefactually reduced activity in some regions during this time. Similarly due to the data storage methods on the EPR used, we may be subject to a degree of false-negative results, whereby results have been received but not yet uploaded to the system.

## Conclusion

This study provides the first assessment of IRDs in Scotland over a 5 year period. We demonstrate that retinitis pigmentosa followed by Stargardt disease were the most common clinical diagnoses, with *ABCA4* found to be the most commonly affected gene. Syndromic retinitis pigmentosa accounted for 15.5% of RP cases, with Usher syndrome the most common cause. Different regions adopted slightly different testing strategies with varying results and time to diagnosis. This work provides a unique insight into the Scottish ophthalmic genetics service and may serve as a benchmark for ongoing improvement in this area.

## Summary

### What was known before:

- Inherited Retinal Diseases are the largest cause of working age blindness in England and Wales.
- Proportion of molecular diagnosis have increased over the years as molecular testing has iteratively improved.

### What this study adds:

- In Scotland, retinitis pigmentosa followed by Stargardt disease were the most common clinical diagnoses, with *ABCA4* found to be the most commonly affected gene.
- Different regions adopted different testing strategies with varying results and time to molecular diagnosis.
- This work provides a unique insight into the Scottish ophthalmic genetics service and serves as a benchmark for ongoing improvement in this area.

## Supplementary information

Supplementary Table S1

Supplementary Table S2

Supplementary Table S3

Supplementary Table S4

Supplementary Table Data

## Data Availability

Datasets analysed in this study are available on reasonable request.
