# Supplementary material for: Inherited retinal disorders in Scotland: A 5 year assessment
Source: Eye (Lond). 2026 Jan 9;40(4):487–92. doi: 10.1038/s41433-025-04216-z (PMC12957440; doi:10.1038/s41433-025-04216-z)
Supplement: Supplementary file 5 — Supplementary Table Data [file 41433_2025_4216_MOESM5_ESM.docx]

**Supplementary Table Data**

Table S1 - IRD diagnoses

Table S2 - Scotland-wide testing strategies (Fig 3)

Table S3 - Frequency of identified genes (Fig 4)

Table S4 – Testing strategies and outcomes
